# What drives the demand for drought-resilient sorghum varieties? Evidence from moisture-stressed areas in Ethiopia

**DOI:** 10.1371/journal.pone.0315985

**Published:** 2025-09-05

**Authors:** Mekonnen Sime, Dereje Mersha, Mesay Yami

**Affiliations:** 1 Ethiopian Institute of Agricultural Research (EIAR), Addis Ababa, Ethiopia; 2 International Institute of Tropical Agriculture (IITA), Ibadan, Nigeria; Fort Valley State University, UNITED STATES OF AMERICA

## Abstract

Sorghum is one of the critical food security crops, particularly in moisture-stressed areas of Ethiopia. However, in the absence of a well-organized formal seed system, public research institutions have continued to promote and disseminate improved sorghum varieties to encourage adoption. On the other hand, the lack of evidence on smallholder farmers’ demand for improved varieties has discouraged the seed industry from investing in marginalized crops, like sorghum, in contrast to more commercialized crops such as wheat and maize. This study assessed producers’ willingness to pay (WTP) for improved sorghum varieties suitable for moisture-stressed sorghum growing agro-ecologies. Data were collected from 659 households selected using probability proportional to size (PPS) sampling techniques. Descriptive statistics, heterogeneity analysis and generalized ordered probit econometric model were employed for data analysis. Farmers’ WTP was, on average, 59% higher than the market price set by the government. In the Amhara and Oromia regions, WTP was 67% and 47% above the official price, respectively. WTP varied significantly by age, farm size, income source, and gender. The inelastic nature of WTP and the observed gender gap—where only 40% of female-headed households exhibited WTP at the market price compared to 60% of male-headed households—highlight the need for gender-responsive, non-price interventions such as targeted subsidies, smaller input packages, and inclusive extension services to promote equitable access and uptake of improved sorghum varieties.

## Introduction

Sorghum (*Sorghum bicolor*) is the fifth most crucial carbohydrate-rich crop globally, following wheat, maize, rice, and barley [1]. In Africa, Ethiopia ranks fourth in sorghum cultivation area (1.82 million hectares), behind Sudan, Nigeria, and Niger [2]. In Ethiopia, the primary staple food is *injera,* a fermented flatbread, traditionally made from *teff.* However, in recent years, sorghum has increasingly been used in *injera* preparation- alone or blended with *teff* flour – due to the rising cost of *teff* and challenges in meeting growing demand through *teff* production alone.

As sorghum is a climate-resilient crop that produces high biomass per unit volume of water, it is considered a potential crop to address Ethiopia’s food and feed demand. Sustainable adoption of yield-enhancing sorghum production technologies and other complementary good agricultural practices (GAP) is critical for transforming agriculture and nutrition in developing countries such as Ethiopia. However, the production area, production, and productivity of sorghum have been declining recently. The current productivity, 2.63 tons/ha [2], is below the national potential of up to six tons/ha. Previous studies have reported 11–37% adoption of improved sorghum varieties in Ethiopia [3–6].

Despite the growing relevance of sorghum as a climate-resilient crop with dual-purpose value (food and fodder), the formal seed system produces a limited quantity of sorghum seeds, which are rarely accessible to smallholder farmers, typically distributed through government programs aimed at specific initiatives [7]. This further resulted in low productivity and food insecurity. The limited supply performance of the supply side resulted from the limited availability of information on the market and demand for sorghum varieties by smallholders.

Empirical research on smallholder farmers’ willingness to pay (WTP) for improved crop varieties has largely focused on staple cereals such as maize, wheat, and rice and associated farm inputs such as mechanization [8–12]. These studies have shaped substantial policy attention and spurred private-sector engagement in the seed systems. In contrast, underutilized crops like sorghum—despite their strategic role in dryland food systems—remain relatively neglected in both seed market development and WTP-based demand assessments. For instance, [13] reported that the information gap on the demand for improved sorghum varieties and smallholders’ WTP contributed significantly to marketing challenges of improved varieties. In a situation with limited information on the market potential for the improved crop varieties, commercialization becomes a challenge, if not entirely impossible [14]. This neglect contributes to the underdevelopment of Ethiopia’s sorghum seed sector, characterized by low varietal turnover, limited private investment, and poor demand-side market intelligence [7,13].

Farmers’ WTP for agriculture innovations and services has significant implications for agriculture development, even at times when access to free services and innovations is constrained by different factors [15]. A few recent studies have begun to assess demand for sorghum varietal traits (e.g., [16,17]), offering valuable input for breeding priorities. However, these studies primarily focused on trait-level preferences without disaggregating WTP by sex, age, or economic characteristics, thereby limiting their relevance for policy-focused interventions. To our knowledge, only [12] have employed a heterogeneity analysis, but their work focused on WTP for maize mechanization services rather than improved sorghum varieties. This study fills a critical gap by generating disaggregated WTP estimates for improved sorghum varieties in Ethiopia using a double-bounded contingent valuation approach. Decomposing WTP across sex, age groups (including youth), landholding size status, and access to non-farm income offers critical guidance for inclusive policy targeting, tailored seed marketing strategies, and varietal development that reflects both the agronomic needs and economic constraints of farmers in moisture-stressed areas of the country.

The remainder of the article is structured as follows: The methods section describes the data collection procedures and empirical strategy used to estimate WTP. This is followed by the results section, which presents descriptive statistics, robustness checks, and key findings from the empirical analysis. The article concludes with the conclusion and policy implications section, which summarizes the main findings and oulines their relevance for policyMethods

### Data and source

The study sites and respondents were selected through a multistage sampling process that combined purposive and random sampling methods. The survey was conducted in seven districts selected purposively, known for drought-proneness and sorghum cultivation in Ethiopia (Table 1).

**Table 1:** Sample size and distribution by district.

Region	District	Number of respondents	Percent
Amhara	Kobo	151	23.03
	Habru	125	18.94
	Kalu	118	17.88
Oromia	Fedis	62	9.39
	Babile	72	10.91
	Gololcha	86	13.03
	Shenenkolu	45	6.82
Total		659	100

**Fig 1:**
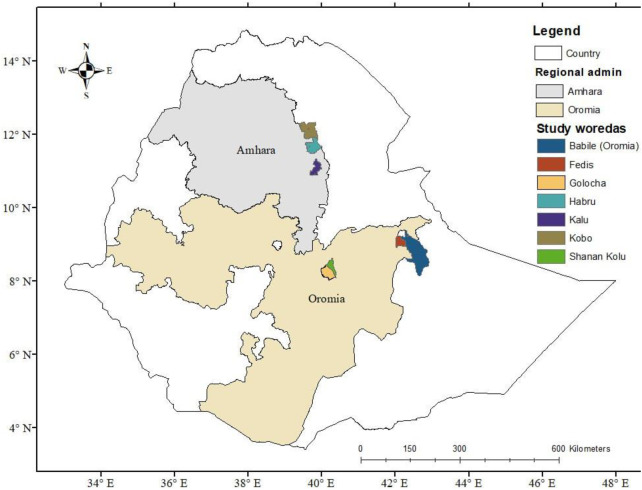
Map showing study areas in Oromia and Amhara regions. This map was redrawn and adapted from [18] for illustrative purposes only.

Regions, districts, and sub-districts (kebeles) were deliberately selected based on the current status and potential of sorghum production. Based on [2], the two regional states, Amhara and Oromia, were selected for the study. The two regions together represent over 74% of sorghum production areas in the country [2]. In Oromia, four districts—Babile, Fedis, Gololcha, and Shenen-Kolu—were chosen, while in Amhara, the districts of Kobo, Habru, and Kalu were selected (Fig 1).

The selected districts fall within a low-potential, low-moisture, and risk-prone agroecological zone (semi-arid) characterized by limited and unpredictable rainfall. This erratic rainfall significantly affects both crop and livestock production. The farming system in these areas is primarily subsistence-based, involving mixed crop-livestock practices, with livestock ownership playing a crucial role in rural livelihoods.

The probability proportional to size (PPS) sampling procedure was followed to identify respondent households. The total sample size was determined using the Cochran formula [19], see equation 1:


$$n_{0}=\frac{Z^{2}pq}{e^{2}}$$ (1)

$n_{0}$ is the sample size, Z-score is the area under the acceptance region in a normal distribution (Z = 1.96) for the 95% confidence interval, squared margin of error (e = 3.8%), the estimated proportion of an attribute that is present in the population (p = 0.5), and q is 1-p. Data were collected from a total of 659 respondent households and 1,598 plots (Table 1).

A structured questionnaire was designed and pre-tested to ensure its validity under local conditions and appropriateness for the target population. The pre-test aimed to assess farmers’ comprehension and ensure that enumerators could accurately translate questions to minimize potential translation bias. The results from the pre-test were thoroughly reviewed, and the questionnaire was revised accordingly, with particular attention given to the core components of the double-bounded contingent valuation (DBCV) method to support accurate economic valuation.

The survey was conducted under the close supervision of researchers from the Ethiopian Institute of Agricultural Research (EIAR). Data were collected using computer-assisted personal interviews (CAPI), a face-to-face technique that enhances data quality and reduces entry errors. Trained enumerators—fluent in the local languages and experienced in agricultural household surveys—were recruited to carry out the interviews. Enumerator training took place in May 2021, and data collection was completed in July 2021. Prior to each interview, enumerators explained the study’s objectives and assured respondents of the confidentiality of their responses. Informed consent was then obtained before proceeding with the interviews.

The data collection was conducted prior to the establishment of the Ethiopian Institute of Agricultural Research (EIAR) Institutional Review Board (IRB) or ethics committee. Consequently, the survey did not undergo formal IRB approval at the time of implementation. However, the study adhered to the rigorous standards and best practices of international agricultural research during data collection. Ethical considerations were integrated throughout the process, including obtaining informed consent from participants and ensuring confidentiality in handling survey data. The study also received clearance for publication from the newly established publication unit of EIAR. Expert feedback was incorporated from the proposal development stage, with the survey proposal reviewed by both the socioeconomics unit and EIAR research management. The proposal was presented and approved at both the center and national levels during proposal review stages, ensuring adherence to institutional guidelines and relevance to national research priorities.

## Method of data analysis

The study employed descriptive, heterogeneity and econometric data analysis methods. Before running the analysis, rigorous data cleaning and an omitted variables test (using the Wald test) were conducted to ensure consistent model estimation.

### Conceptual framework

**Fig 2:**
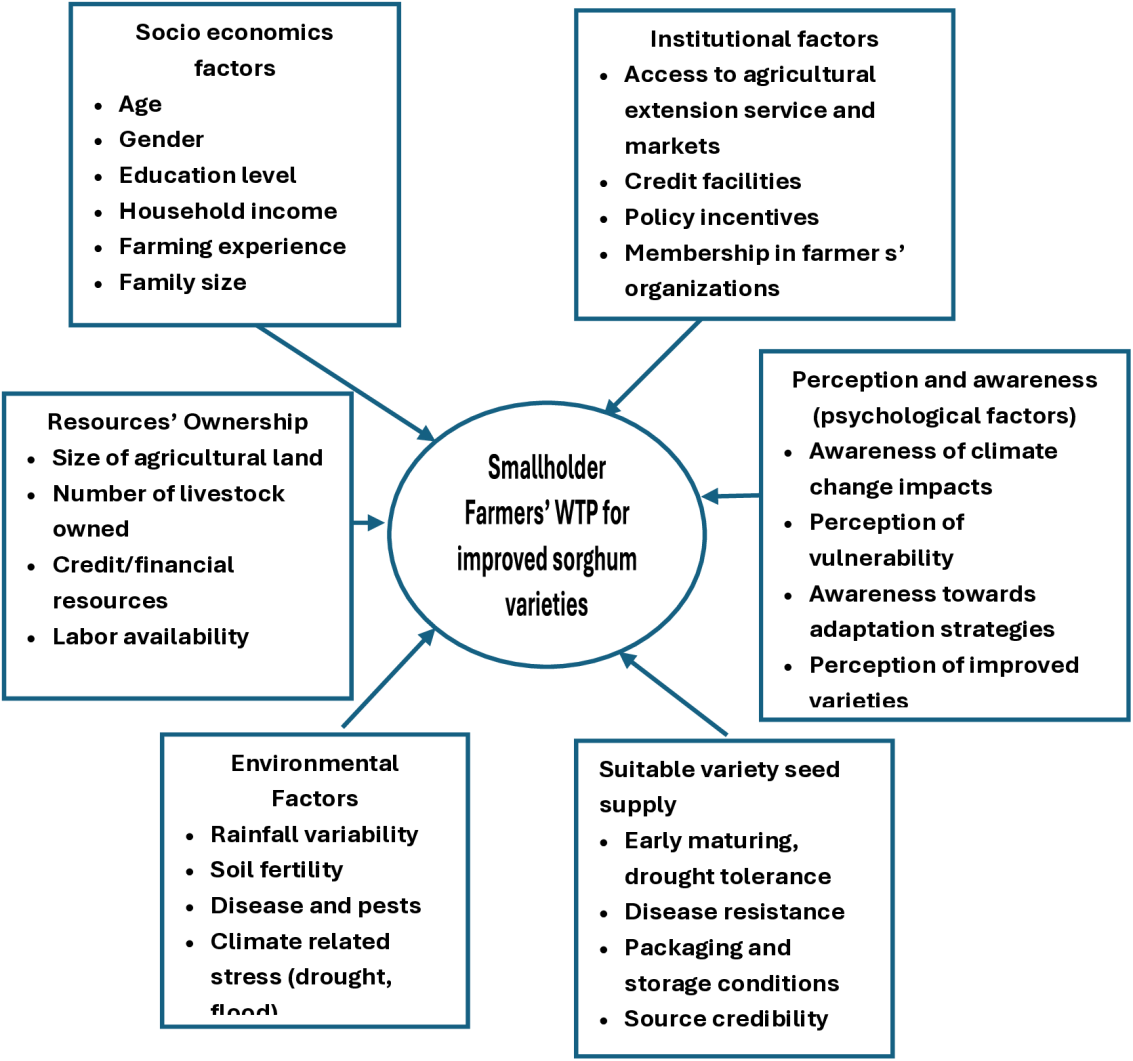
Conceptual framework of smallholders’ WTP for improved sorghum varieties.

Understanding the WTP of smallholder farmers for improved sorghum varieties is critical for demand-led innovation, price planning, and seed system development. WTP studies seek to understand how much farmers pay for certain features. Conceptually, WTP is framed under utility theory where farmers balance the marginal utility of improved attributes (e.g., drought tolerance, yield, early maturity) and the opportunity cost of their acquisition. Empirical studies indicated socioeconomic, resource endowments, institutional, perception/awareness of technologies, environmental, and suitability of improved varieties to the production environments, factors influencing farmers’ WTP for improved varieties (Fig 2 below).

A study from Ethiopia indicated that sex (male), education level, extension contact, on-farm income, and credit significantly and positively influence smallholder farmers’ WTP for improved tef varieties [20]. Resource endowment of household (e.g., land and livestock), and farming experience were found to influence farmers’ WTP for improved crop varieties in Ethiopia [21]. Another study from Nigeria highlighted that age, farming experience, farm size, and farm income influence farmers’ WTP for improved pigeon pea variety [22]. Institutional support, such as access to extension services was found to positively influence farmers’ WTP for improved varieties [8].

Aversion to risk among farmers had a negative influence on WTP for both technologies ( [8]. The results suggest significant willingness to sacrifice yield in favor of drought tolerance among both male and female members in a household [23]. The same study revealed that significant willingness to sacrifice yields in favor of plant architecture traits like closed tip and lodging resistance among males. Household head educational level, cultivated land holding size, agriculture training participation, extension agent contact, and market price had positive and significant effects on the probability of WTP, while farm worker possession, i.e., economically active household member number and location-to-road head distance had negative effects. Accordingly, the conceptual framework of WTP for improved sorghum varieties is examined under these dimensions.

### Analytical framework

#### Contingent valuation method

The existing array of literature indicates two theoretical approaches being used to estimate WTP. The two valuation approaches, direct and indirect, are commonly used for non-marketed goods ([24,25]). As indicated by [24], the direct methods try to elicit information on the value of the non-marketed goods/services directly from the individual, while the indirect estimations are based on the observed behavior of individuals in the market of a good or service related to one’s interest.

Contingent valuation model (CVM), which was originally used in environmental and resource economics, was also employed to elicit farmers’ demand for improved crop varieties ( [26–28]. This study also employed the approach to elicit the demand for improved sorghum varieties suitable to moisture stressed areas. CVM involves asking a respondent about his/her WTP for a given good or service [24]. Unlike the experimental actions where the goods are demonstrated and people commit themselves economically, the CVM (contingent upon a constructed or simulated market) asks respondents to choose between options [29].

WTP can be estimated either with open-ended questions, like asking respondents the amount they are WTP, or closed-ended questions, like asking if they would be WTP a specified amount (dichotomous option) [26]. Following [30] and [24], this study employed a double-bound dichotomous choice (DBDC) CVM to measure smallholder farmers’ WTP for the preferred sorghum varieties. The DBDC is preferred over single-bounded due to its statistical efficiency. In the single-bounded case, the individuals provide very limited information regarding WTP [24]. With the DBDC approach, the respondent’s response for the stated amount of money is expected to be” yes” or ”no” and then to be asked a follow-up question to say “yes” or “no” for the higher or lower bids [[12,24,30–32]].

Although the stated preference approach, DBCV approach has been criticized for biased estimation, the bias is much lower when responses are based on marketable goods than on non-marketable goods [8]. [33], indicated that the bias of CVM can be minimized using an appropriate survey design. Following [8], a realistic bid range was introduced to minimize the unrealistic bid price range that leads to starting bias in the DBCV approach. Besides, like [34], the study used sorghum improved varieties that are actual and known in farmers’ environment.

#### Empirical estimation

Farmers were asked whether they would be WTP for an improved sorghum variety with a “yes” or “no” question relative to improved sorghum seed market price (27.6 Ethiopian Birr (ETB) per kg) set by the Ministry of Agriculture (MoA)/national seed committee [one ETB was equivalent to ~ 0.025 USD during the survey year]. If the response was “yes” or “no” they were further presented with the follow-up WTP questions with a higher or lower bid price of the initial bid (50% more or 50% less than the market price).

**Fig 3:**
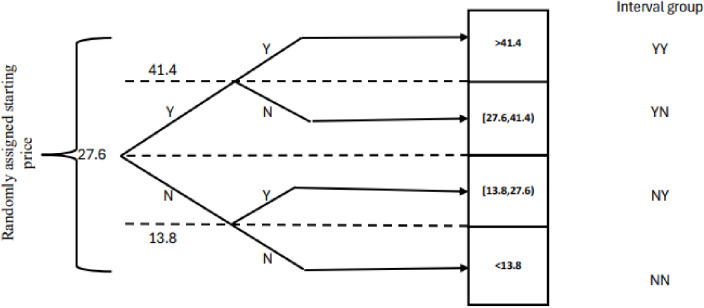
Structure of DBDC experiment design for sorghum seed. Note Y=Yes, N=No; Adapted from [8].

Based on their WTP or the level of offer, respondents were categorized into four groups (Fig 3): the highest bidder (≥ 41.4), high bidder (27.6, 41.44), low bidder (13.8, 27.6) and lowest bidder (≤ 13.8).

Accordingly, ordered probit or logistic models are more appropriate for analysis of such ordered response variables [35–37]. The logistic and probit models yield similar results in practice (see [8]). The model assumes the latent variable framework, an unobservable and unmeasurable variable. This latent variable, respondents’ choice/WTP, is a function of observable explanatory variables such as socioeconomic characteristics [38]. Based on [37], let y be the ordered response, taking the values {0, 1, 2, …J} for some known integer J. The ordered probit model for y (conditional on explanatory variables x) can be derived from the latent variable model. Assume that the latent variable $y^{*}$ is determined by


$$\begin{array}{c} y^{*}=x^{,}\beta +e,\;e|x\;\sim Normal\;(0,1) \end{array}$$ (2)

where $\begin{array}{c} \beta \;is\;K\;X\;1 \end{array}$ is a reason to be seen doesn’t contain a constant. Let $\alpha_{1}<\alpha_{2}<\alpha_{J}$ be unknown cut points (threshold parameter), and define


$$\begin{array}{c} \begin{array}{c} y=0\;if\;y^{*}<\alpha_{1}\\ y=1\;if\;\alpha_{1}<y^{*}<\alpha_{2}\\ \;.\\ \;.\\ \;.\\ y=J\;if\;y^{*}>\alpha_{J} \end{array} \end{array}$$ (3)

J is a mutually exclusive categories of $y_{i}$, 1≥i≤J. If $y_{i}$ takes on the values 0, 1, and 2, then there are two cut points, $\alpha_{1}$ and $\alpha_{2}$

Given the standard normal assumption e, it is straightforward to drive the conditional distribution of y given x; we compute response of each probability:


$$\begin{array}{c} \begin{array}{c} \mathrm{Pr}ob(y=0|x)=p(y^{*}\leq \alpha_{1}|x)=p(x\beta +e\leq \alpha_{1})\Phi (\alpha_{1}-x^{\prime }\beta )\\ \mathrm{Pr}ob(y=1|x)=p(\alpha_{1}<y^{*}\leq \alpha_{1}|x)=\Phi (\alpha_{2}-x\beta )-\Phi (\alpha_{1}-x\beta )\\ \;.\\ \;.\\ \;.\\ \mathrm{Pr}ob(y=J-1|x)=p(\alpha_{J-1}<y^{*}\leq \alpha_{J}|x)=\Phi (\alpha_{J}-x\beta )-\Phi (\alpha_{J-1}-x\beta )\\ \mathrm{Pr}ob(y=J|x)=p(y^{*}>\alpha_{J}|x)=1-\Phi (\alpha_{J}-x\beta ) \end{array} \end{array}$$ (4)

It can be verified that this can be sum to unit. When J = 1 we get the binary probit model: $p(y=1|x)=1-\Phi (\alpha_{1}-x\beta )=\Phi (x\beta -\alpha_{1})$, and so $-\alpha_{1}$ is the intercept in Φ. It is for this reason x that it doesn’t contain an intercept in this formulation of the ordered probit model. When there are only two outcomes, zero and one, we set a single cut point to zero and intercept; this approach leads to a standard probit model for the intercept; the parameters $\alpha_{1}and\;\beta$ can be estimated by maximum likelihood. For each i, the log-likelihood function is


$$\begin{array}{c} li(\alpha ,\beta )=[y_{i}=0log[\Phi (\alpha_{i}-x_{i}\beta )]+1[y_{i}=1]\log\Phi [(\alpha_{2}-x_{i}\beta )\\ -\Phi [(\alpha_{1}-x_{i}\beta )+...+1[y_{i}=J]\log[1-\Phi (\alpha_{J}-x_{i}\beta ) \end{array}$$ (5)

This log-likelihood is well-behaved, and many statistical packages routinely estimate ordered probit models [37]. Ordered logit model can be estimated by replacing Φ by Λ. It should be noted that β by itself is of limited interest in either case. In most cases, $\left(y^{*}=x^{,}\beta \right)$, as $y^{*}$ is an abstract construct [37].

However, the parallel regression assumption of homogenous effect of coefficient vector β is assumed to be the same for all categories J: the four states of WTP (lowest to highest) is one of the limitations of the standard ordered probit model [39]. One way of testing the parallel regression assumption is by comparing the estimates of standard and generalized ordered probit regression models. In the generalized ordered response model, the thresholds are allowed to vary by individuals in a way that individual heterogeneity can be modeled through allowing thresholds to be a function of variables that condition category probability and leading to efficient estimation [40]. Following [40], we estimated the Skewed Generalized T distribution (SGT) which was introduced by Theodossiou (1998 as cited in [40]). The SGT nests other commonly used distributions to accommodate a wider range of skewness and kurtosis. The SGT commutative distribution is given by:


$$SGT(\varepsilon ,m,\lambda ,\sigma pq)=\left(\frac{1-\lambda }{2}\right)+\frac{1+\lambda sign(\varepsilon -m)}{2}sign(\varepsilon -m)B_{z}(1/p,q)$$ (6)

where the incomplete beta is represented by $B_{z}$ and is given by


$$z=\frac{|\varepsilon -m{|}^{p}}{|\varepsilon -m{|}^{p}+q\sigma^{p}{\left(\lambda sign(\varepsilon -m)\right)}^{p}}$$ (7)

where parameters m and k represent location and control skewness while q are positive shape parameters that determine skewness and kurtosis. Allowing the parameter q in the SGT to grow indefinitely large results in a skewed generalized error distribution (SGED). The cumulative distribution for the SGED is indicated as follows.


$$SGED(\varepsilon ,p,\lambda ,p)=\frac{\left(1-\lambda \right)}{2}+\frac{\left(1+\lambda sign(\varepsilon -m)\right)}{2}sign(\varepsilon -m)\Gamma_{z}(1/p)$$ (8)

where $\Gamma_{z}$ is the incomplete gama function and,


$$z=\frac{|\varepsilon -m{|}^{p}}{\sigma^{p}{\left(1+\lambda sign(\varepsilon -m)\right)}^{p}}$$ (9)

The distributions are symmetric if λ=0 at which the SGT and the SGED yielding the generalized (GT) introduced by [41] and generalized error distribution (GED), respectively. Letting p = 2 in the SGT yields the skewed (ST) [42]. To find the Skewed Laplace (SLaplace), set in the SGED and to get the skewed normal (SNormal) set. The distributions modeled in this paper are the SGT, SGED, SNormal, GED, SLaplace, Laplace, and Normal which are selected following careful analysis of some of the distributions of the SGT distribution. They are some of the more general distributions, and they give the results allowing to compare the advantages of relaxing different parameterization assumptions.

#### Variables and descriptive statistics

As indicated in Table 2 below, 94% of the respondents were male, while the remaining were female-headed households. The average age and family size of the respondents were 43.7 and 5.78. The average farm size owned and operated areas were 1.2 ha and 1.5 ha, respectively, indicating the area is dominated with smallholder and subsistence production. Of the total operated area, 0.71 ha was allocated for sorghum with a 1916.53 kg/ha productivity level. The households also own assets valued at 47590.4 Birr on average. Livestock and crop production are integrated activities in the area with a 3.3 tropical livestock unit (TLU), although lower than the national average (4.46 TLU). Income is one of the most powerful determinants of individuals’ WTP for improved agricultural technologies. In most instances, households or individuals with greater incomes have greater capacity and WTP because they have greater disposable income to spend on perceived beneficial products. In this study, household income was calculated from annual livestock/livestock product sales, crop, and non-farm incomes. As indicated in Table 2, the income of respondent households in the area was 103,575 ETB, on average, with high variability.

**Table 2:** Description and descriptive statistics of variables.

Variables and definition/note	Expected signs	Mean (%)	Standard deviation
Age of the household head (years)	+/-	43.7	12.1
Gender of household head (1 = Male)	+	94	–
The education level of the household head (years of schooling)	+	3.0	3.3
Sorghum production experience of the household in years	+/-	22.4	11.2
Family size of the household (number)	+/-	5.8	2.2
Household total income in ETB (logincome)	+_	103575	179469
Total plot area operated by the household (ha)	+	1.5	1
Membership of household head in leadership position (1 = yes)	+	21.0	–
Household who used credit (1 = yes)	+	19.2	–
Total asset value (ln ETB)	+	47590.4	40619.1
Received training on climate change and adaptation (1 = yes)	+	23.36	
Distance to extension service providing office (kilometer/km))	–	2.4	1.5
Distance to input markets (km)	–	6.6	4.5
Plot distance from residence (km)	+/-	1.8	1.4

Source: Computed from survey data (2021).

Trainings often help to enhance farmers’ awareness, knowledge, and perception of the agronomic and economic benefits of drought-tolerant varieties. Participation in climate change and adaptation training was expected to significantly influence smallholder farmers’ WTP for improved sorghum varieties adapted to low moisture areas. Accordingly, farmers who have attended such training are more likely to recognize the long-term advantages of adopting improved seeds, such as increased yield stability, better risk management, and resilience to climate variability—thereby demonstrating a higher WTP.

## Results and discussion

### What explains the WTP for improved sorghum variety?

In the study areas, over 77% of respondents reported that their own saved seed is dominantly used, and sorghum seed is being recycled for about 9.7 years, on average. Of the 660 households, 35% used improved sorghum varieties in the 2020 cropping season. In recent years, smallholder farmers were introduced to different improved sorghum varieties though on farm variety evaluation and demonstrations. During the evaluation, farmers were requested to set their selection criteria (preferred traits) in identifying the variety that suits their context. Table 3 below presents farmers’ sorghum trait preference criteria, showing the most important traits in sorghum varieties, with early maturity (mean score: 4.05) and ranked first. This indicates that farmers prefer varieties that can mature early, likely due to the importance of minimizing risk in locations with shorter growing seasons or prone to drought. Grain yield (3.87) and grain color (3.81) are key traits next to early maturity, reflecting the emphasis on productivity and marketability. Farmers prioritize these traits to maximize their returns, either through higher yields or meeting market preferences through grain color. Grain size (3.80) and palatability of the stalk (3.64) are also important.

**Table 3:** Farmers’ sorghum traits preference (N = 426).

Variety characteristics	Mean score	Rank
Grain yield	3.87	2
Stover yield	3.31	11
Palatability	3.64	5
Early maturity	4.05	1
Grain color	3.81	3
Cooking time	3.58	7
Grain size	3.80	4
Drought tolerance	3.57	8
Disease tolerance	3.39	10
Insect pest tolerance	3.25	12
Storability	3.41	9
Taste	3.59	6

Remark: 1= Very poor 2= Poor 3= Average 4= Good5= Very good.

The preference for taste (3.59) and storability (3.41) indicates the importance of sorghum as a staple food crop, with the long-period storability playing a critical role in ensuring household food security. These traits suggest that farmers prioritize not only the yield and quality of sorghum but also its use as a dependable food source, especially in regions with inadequate access to storage facilities or erratic harvest cycles.

**Fig 4:**
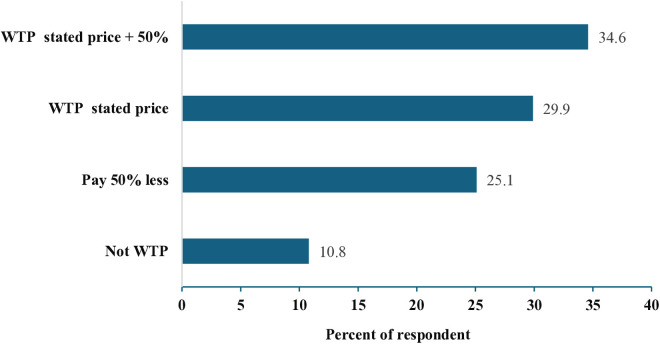
Respondents’ distribution by WTP group (n = 657).

Based on the preferred traits discussed above, farmers’ WTP for improved sorghum varieties was measured in Ethiopian Birr (ETB/kg). Fig 4 shows the distribution of respondents by their WTP level. Among the total survey participants, 34.55% were WTP the highest price (the price set by MoA plus 50%), while 29.53% were WTP the current price set by MoA. On the other hand, 25.11% of respondents indicated they would only pay if the price were at least 50% lower than the current price. Meanwhile, 10.81% were not WTP for seeds of improved sorghum varieties at all. Most respondents (64.08%) were WTP at least the current price, while 35.92% preferred prices below the current market level for improved sorghum seeds.

### Determinants of WTP: Econometric estimation

During the model specification phase, 5 of the 14 variables described in Table 3 that did not contribute to an optimal model fit were excluded to enhance the model’s overall performance. Hence, model estimation was conducted using nine variables. Before the estimation of generalized ordered probit, a formal test of the generalized against the ordered probit model was conducted using the wald test. The overall Wald test statistics of generalized over standard probit indicates that at least one predictor violates the proportional odds assumption (each predictor is identical across all outcome categories at Chi2 (27) = 128.17, p < 0.000. Furthermore, a wald test was applied on each variable to identify variables’ distributional impacts, and it was found that the null hypothesis of equal coefficients was rejected in seven of the nine variables (except family size and distance to market) at 5 percent level of significance. This validates the use of generalized over the standard ordered probit. Generalized ordinal probit regression analysis was carried out to find out the main factors influencing farmers’ WTP for the improved sorghum varieties. The slope coefficient and Z-value estimates from generalized ordered probit model estimation is presented in Table 4 below. The Chi-squared is statistically significant at Chi2(27) = 136.07, p = 000) at less than 1% significance level, indicating that the joint test of all slope coefficients equal to zero is rejected.

**Table 4:** Generalized ordered probit estimation of WTP (n = 654).

Variable	Threshold 1 p(y>1)vs.y=1	Threshold 2 p(y>2)vs.y≤2	Threshold 3 p(y>3)vs.y≤3
Gender	0.874 (0.248) ***	0.863 (0.224) ***	0.565 (0.254) **
Age	0.025 (0.007) ***	0.015 (0.005) ***	0.002 (0.005)
Family size	−0.121 (0.035) ***	−0.057 (0.026) **	−0.022 (0.026)
Total land operated	0.080 (0.081)	0.129 (0.053) **	0.155(0.051) ***
Income	−0.142 (0.085) *	−0.107 (0.069)	−0.096 (0.070)
Asset value	0.147 (0.053) ***	0.063 (0.041)	0.074 (0.043) *
Market distance	0.007 (0.015)	0.019 (0.012)	0.020 (0.012) *
Received training on climate change adaption	0.323 (0.198) *	0.642 (0.136) ***	0.563 (0.123) ***
Plot distance	0.224 (0.061) ***	0.080 (0.040) **	0.078 (0.038) **

Note: numbers out of parenthesis representing coefficients, numbers in parenthesis representing standard errors, *** = significant at 1% probability level ** = significant at 5% probability level * = significant at 10% probability level.

As indicated in Table 4 below, being male household increases the likelihood of being in the higher thresholds as compared to female household. The influence is statistically significant at 1% for threshold 1 and threshold 2 and moderately significant at the highest category. The result is in line with the findings of other studies conducted in Ethiopia and Kenya, which reported gender (being male) positively influences smallholder farmers’ WTP for improved technologies (seed or fertilizer) [8,20,43].

A unit change (year) in age also increases the likelihood of avoiding being in the lowest category at 1% significance level and similarly increases the likelihood of advancing to higher category (being in category three or four) at 1% significance level showing a declining influence. The result is in line with the findings from Nigeria [44] and Ghana [44].

Family size negatively influences the likelihood of being in the higher category at 1% significance level at thresholds 1 and 5% level at threshold 2, implying that the larger the family size, the higher the likelihood of falling into lower WTP categories, indicating that a large family may face resource constraints to meet basic needs. The result agrees with the study on smallholders’ WTP for improved varieties of soybeans in Kenya [45]. The total land operated farm size of sorghum growers shows a positive and strongly significant influence (5% significance level) on the likelihood of moving to a higher WTP category at threshold 2, and becomes even stronger and highly significant (1% level) at threshold 3. The result entails that large farmland size increases the likelihood of belonging to the highest WTP categories, which is in line with findings of other countries [[15,20,34]].

Interestingly, income has shown a negative and weak influence on the WTP for improved sorghum varieties. It is only significant at threshold 1, indicating the influence is negative and marginally significant at a 10% significance level. This could imply that sorghum is less prioritized by higher-income groups or less attractive for commercialization. Unlike income variable, asset value showed positive influence on thresholds 1 and 3 at 1 and 10% significance levels, respectively. The influence of market distance is positive and only significant at threshold 3 and at 10% significance level, implying that farmers at remote locations are more likely to be in the top WTP category. This could be due to the fact that the majority of sorghum is located in far and harsh production environments.

Participation on climate change and adaptation training has a positive and progressively increasing influence across the three thresholds at less than 10% for threshold 1; and 1% for threshold 2 and threshold 3 suggesting that training significantly enhances sorghum growers’ WTP for improved sorghum varieties with preferred traits such as drought escaping early maturing variety. The result is in line with a similar WTP study for improved soya bean seed and rice [[8,46]]. The result underscores the importance of agricultural training in enhancing farmers’ knowledge, skills, and adoption of best practices.

The distance from the farmers’ plot to their residence has shown a significant and positive effect in all the thresholds, with the strongest effect in threshold 1 (at 1% significance level) and lesser but significant effects in thresholds 2 and 3 at 5% significance levels. This suggests that farmers with plots at a farther distance show a higher likelihood of being in the higher WTP categories. But the diminishing size of the coefficients indicates that the effect is greatest when moving from the lowest WTP category. This is against other studies reporting the negative influence of plot distance on WTP for improved crop varieties [47]. The possible reason could be due to the fact that most of the sorghum plots were located farther from growers’ residences.

### Demand for improved sorghum varieties

**Fig 5:**
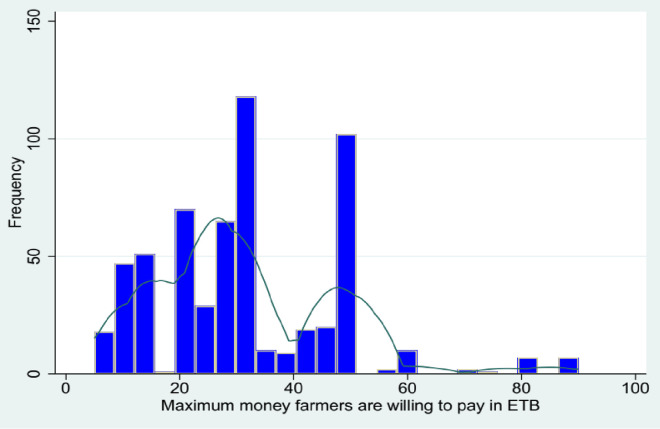
Maximum value smallholder farmers are willing to pay.

Fig 5 shows the WTP distribution, with a mean of 31.36 and a standard deviation of 3. Most observations fall between 20 and 40 ETB per kg.

**Fig 6:**
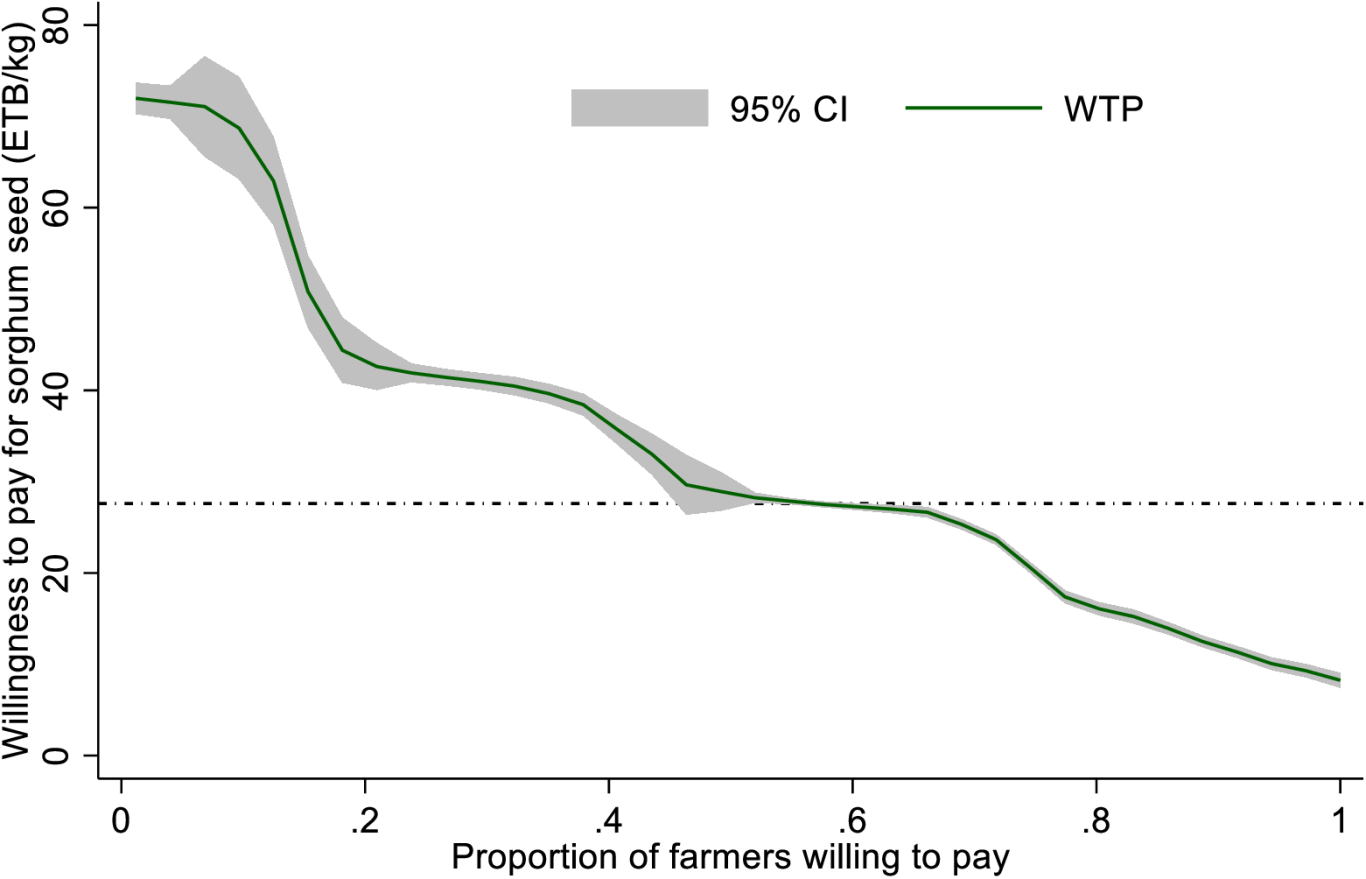
Estimated demand curve for improved sorghum seed in Ethiopia.

As discussed above, MoA set the market price for a kg of the improved sorghum variety at 27.6 ETB during the survey year. Of the 587 respondents, 352 (59%) provided WTP above the market price. We estimated a smoothed version of the demand curve, as shown in Fig 6, to examine the percentage of respondents whose WTP suggests they would pay more than the market price. The result indicates that selling prices must drop to 20 ETB/kg for more than 80% of respondents to purchase and adopt the new sorghum variety.

**Fig 7:**
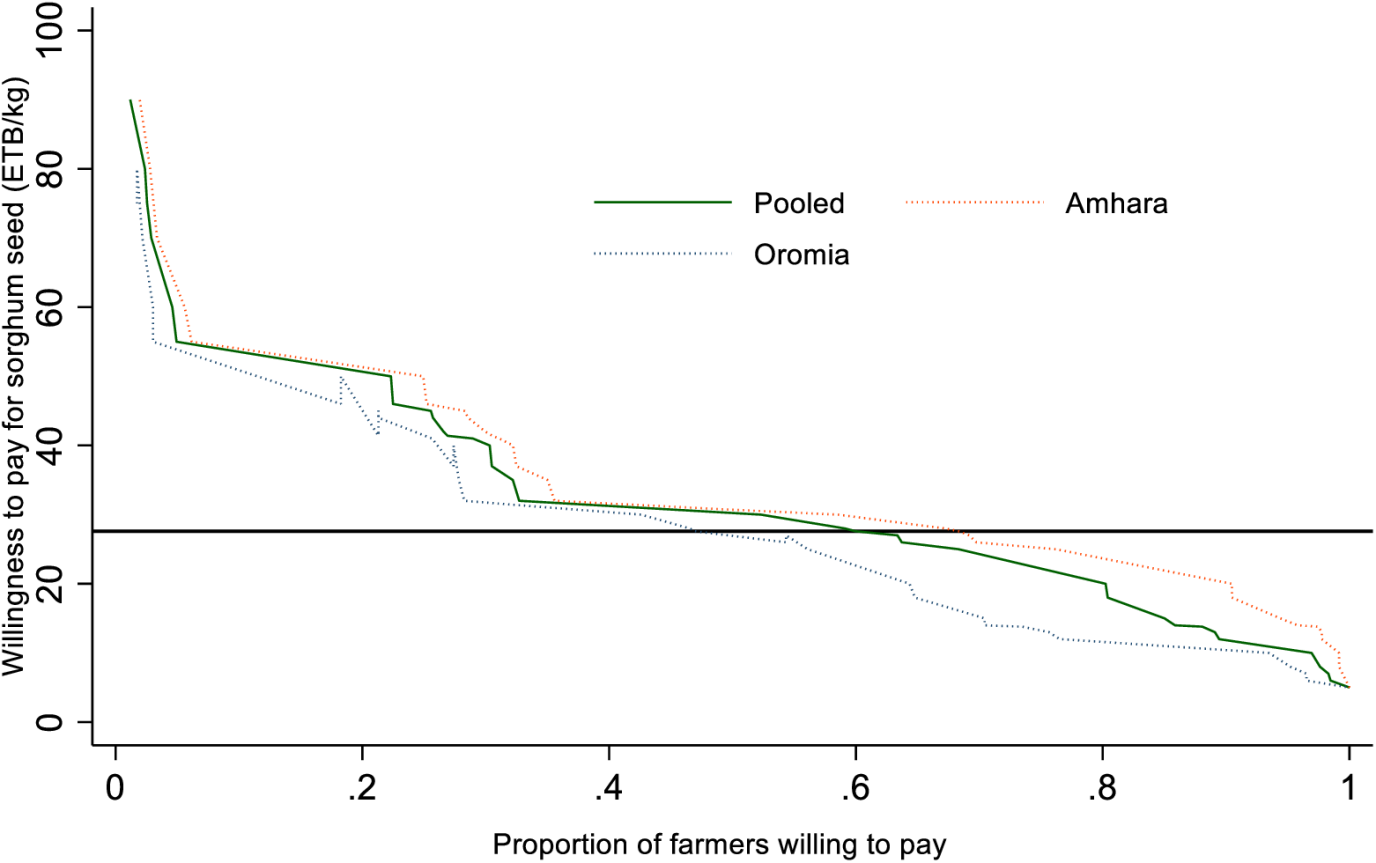
Demand curve for improved sorghum seed by regional state.

Compared to farmers in the Oromia region, farmers in the Amhara region are more willing to pay for improved sorghum seeds (Fig 7). As a result, efforts should be made to increase seed availability in the Amhara region along with effective technology demonstrations. In the Amhara region, more farmers are WTP than the ongoing market rate for the seed. Farmers in the Amhara region were WTP 67% more than the market price for the improved sorghum than farmers in the Oromia region (47%). This can be because of the Oromia region’s proximity to and accessibility of research and seed enterprises. In the Oromia area, research centers under EIAR engage in the multiplication of breeder seeds and the demonstration of new sorghum varieties. Therefore, farmers in the Oromia region may have easier access to sorghum seed and prefer to give lower rates for the crop than in the Amhara region. Farmers in the Amhara region are willing to spend 33.8 birr per kg on average, 23.7% more than farmers in the Oromia region, for the new sorghum seed. The results of this study are consistent with a similar study by [10], which claimed that in Africa’s smallholder agricultural system, the promotion and demonstration of new technology are primarily perceived as gifts. This is partially true in the extension method used in Ethiopia, as farmers who organize demonstration events would be given free ownership of the technology after the trial, which would lessen their incentive to invest in new technology.

**Fig 8:**
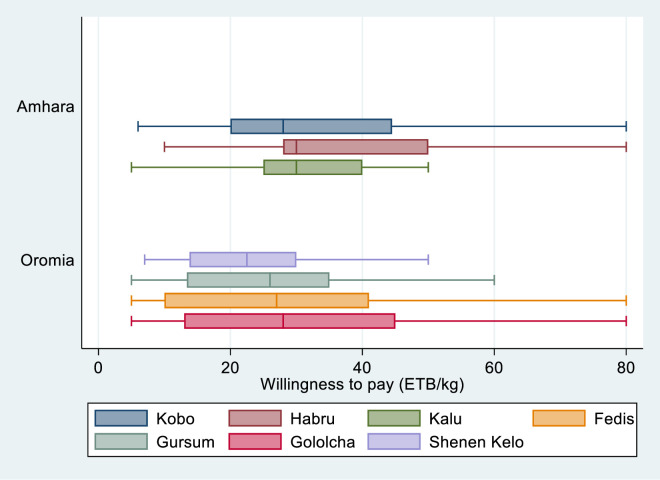
Comparative analysis of WTP by district/region.

Fig 8 below compares WTP made on a district-by-district basis. The WTP for the surveyed districts in the Amhara region is consistently higher than that of the studied districts in the Oromia region. Farmers from the Habru, Kobo, and Kalu districts in the Amhara area were more prepared to pay for new sorghum varieties than farmers from other districts. The price they were WTP ranges from 33 to 38 ETB per kilogram. On the other hand, farmers who were interviewed in the districts of Oromia, specifically Gololocha, Fedis, Gursum, and Shenen Kolo, indicated the lowest WTP on average for the sorghum varieties. This can be explained by the lower value of the product in the Oromia region since most of the areas had access to improved seed and can be recycled for 3–4 years. Otherwise, sorghum is widely favored in both regions, as farmers and urban dwellers blend its flour to produce injera (staple local food) and use it mostly for consumption and other local drinks.

Sorghum is utilized in various ways, and the prices of its alternative commodities, such as teff, have been rising. These findings show that in addition to the value of a commodity, diversities in which the commodity can be utilized are also a key factor in determining WTP. A commodity with a higher household consumption rate and an additional substitution advantage will have a higher competitive edge than its marketability in an imperfect rural output market condition, such as in Africa. As a result, the study offers breeders and seed multipliers relevant information that can assist them in striking a balance in the generation and multiplication of technologies with high-end-use quality, which can support household food consumption and substitute other food commodities in the face of rising food prices. This is especially beneficial for households with limited resources.

The collective demand for improved sorghum varieties for low-moisture stress areas was estimated using expert judgment. [48] reported that approximately 72% of the total area of sorghum cultivation in Ethiopia falls under low-moisture agro-ecologies. With a national area under sorghum cultivation being 1.82 million hectares in 2020 [2], relative annual demand for improved sorghum seed can be approximated as up to 13,119.26 tons (Table 5). This estimate is based on complete varietal substitution using a seed rate of 10 kg per hectare across all low-moisture sorghum production areas. In monetary terms, the total demand was valued at approximately 411,419,994 Ethiopian Birr (ETB) estimated based on average price respondents were willing to pay (31.36 per kg), equivalent to about 10,285,499.84 USD based on the 2021 exchange rate (1 ETB ≈ 0.025 USD).

**Table 5:** Total demand for improved sorghum varieties.

Regional state	Total area planted (ha) in 2020	Area in low moisture stressed areas	Total seed required in tons	Total value of the demand (ETB)
Amhara	641,614	461,962.08	4,619.62	144,871,283
Benishangul/Gumuz	52,006	37,444.32	374.44	11,742,438
Dire Dawa	8,030	5,781.60	57.82	1,813,235
Gambella	3,689	2,656.08	26.56	832,922
Harari	8,030	5,781.60	57.82	1,813,235
Oromia	714,493	514,434.96	5,144.35	161,326,816
Southern Nations, Nationalities and Peoples Representatives (SNNP)	105,256	75,784.32	757.84	23,765,862
Somali	56,365	20,425.63	204.26	6,405,594
Tigray	232,636	167,497.92	1,674.98	52,527,373
Afar	2,765	1,990.80	19.91	624,378
**Total (National)**	**1,822,119.00**	**1,311,925.68**	**13,119.26**	411,419,994

Source: computed from survey data (2021).

### Heterogeneity analysis

**Fig 9:**
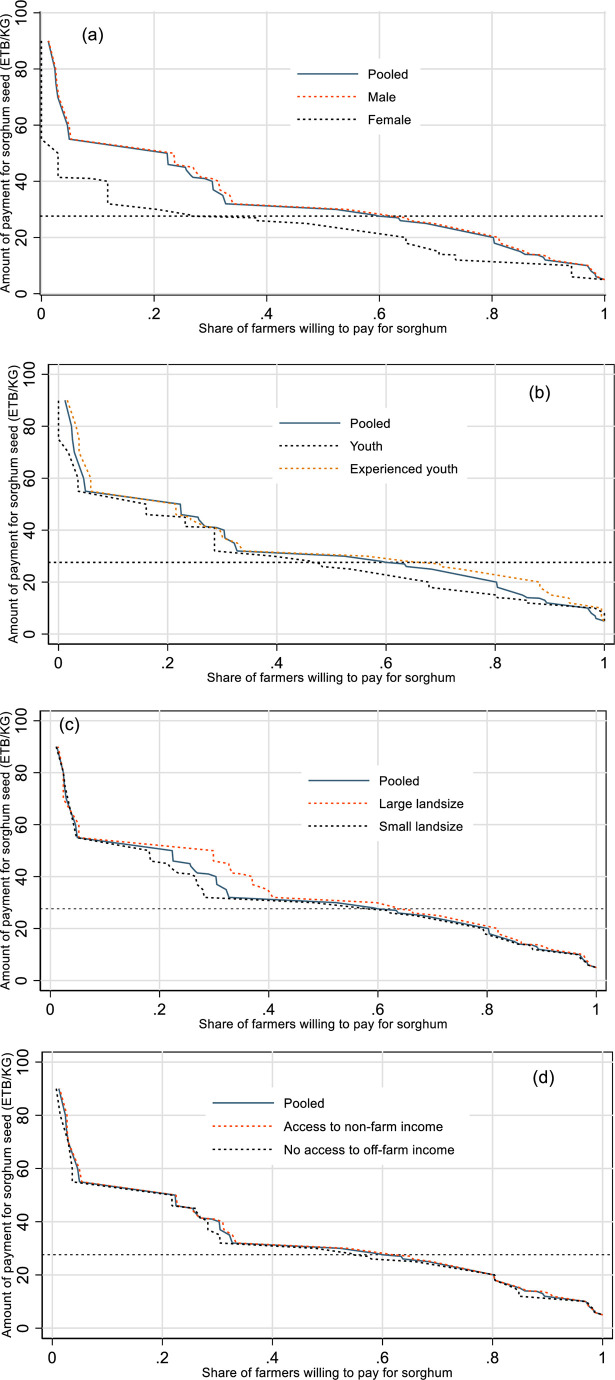
Demand for improved sorghum variety by sex (a), age (b), land size (c), and non-farm income (d).

WTP can vary due to wealth, market access, and agroecology differences [12]. Thus, to design relevant actionable policy recommendations, we further disaggregated WTP for the new sorghum variety by sex, age structure, farm scale, and access to non-farm income. The demand curves in Fig 9 show a decreasing slope and are generally inelastic for all categories, which is consistent with theory and suggests that farmers’ WTP for improved sorghum variety decreases as the bid price rises. Overall, the WTP increases with sex, age, cultivated land size, and non-farm income access.

The heterogeneity analysis (Table 6) reveals a clear gender disparity in WTP for improved sorghum varieties: only 40% of female-headed households were willing to pay the prevailing market price, compared to 60% among male-headed households. While this gender inequality reflects broader patterns observed in various findings in Ethiopia [49], Malawi [12], and India [50], it is not merely a reflection of income or land ownership constraints. Rather, it underscores the structural constraints to nonland based barriers, such as limited access to extension services, information asymmetry, and sociocultural norms that limit women’s participation in agricultural decision-making ([51–53]). We do not attribute the difference in WTP to land constraints as we are comparing male-headed and female-headed households, not intra-household resource allocation, and each managing separate sorghum plots. As explained by Peterman et al. (2014), nonland agricultural inputs include access to agricultural innovations (agrochemicals, improved varieties, and farm equipment), natural resources, and human resources (including extension services).

**Table 6:** Heterogeneity analysis using predicted WTP.

Variable	Observation	Mean	Standard deviation
Sex			
* Male*	553	31.93	16.37
* Female*	34	21.90	11.00
Age structure			
* Youth*	56	27.85	15.31
* Experienced youth*	345	31.10	16.54
* Old youth*	186	32.86	15.95
Farm size			
* Small land size (<=1.52 ha)*	376	30.23	15.98
* Large land size (> 1.52 ha)*	211	33.35	16.62
Family size			
* Low household size (<=5 members)*	289	32.40	16.21
* High household size (>5 members)*	298	30.33	16.29
Non-farm intensification			
* Nonfarm income*	138	30.35	16.03
* No access to nonfarm income*	449	31.66	16.35

In Ethiopia, extension services are predominantly accessed by male farmers, with women often excluded due to mobility constraints, male-dominated extension staff, and gendered assumptions about farming roles ([54,55]). This leads to significant differences in technology adoption rates and WTP between genders. For example, evidence from gender-inclusive extension interventions shows that when women are directly engaged—such as through video-mediated trainings and women-specific groups—their understanding of technologies and willingness to pay improve significantly [[54,56]]. These interventions have also shown that participation of both spouses enhances post-training discussion and adoption decisions, further reducing gender disparities.

Furthermore, asset ownership plays a crucial role in shaping WTP. Women in many developing countries typically control fewer productive assets than men, which limits their ability to invest in improved inputs [12]. Control over assets not only facilitates access to credit but also builds confidence and bargaining power in intra-household decision-making, enabling more permanent pathways out of poverty [57]. Hence, addressing gender-based barriers in both access to information and productive resources is essential for equitable uptake of agricultural innovations. As highlighted by [58], achieving gender parity in access to productive inputs could raise farm yields by 20–30% and substantially reduce food insecurity in developing countries.

## Conclusion and policy implications

This study aims to understand smallholder farmers’ demand for new sorghum varieties in moisture-stressed areas of Ethiopia. Considering the market prices set for improved sorghum varieties by the MoA, excluding other inefficiencies of market fundamentals, the findings from the study suggest that new sorghum varieties adaptable to moisture-stressed conditions can be commercialized with the existing demand. Farmers were WTP 59% more than the set market price by the government. The WTP for farmers in the Amhara and Oromia regions was 67% and 47% more than the determined selling prices for the newly released sorghum variety. The low varietal turnover could be challenging in encouraging private sector seed businesses, where most farmers relied on their farm-saved seeds (70%). This calls for enhancing farmers’ awareness through sensitization on the importance of seed replacement and the use of improved seed.

Farmers showed a strong preference for sorghum varieties that are early maturing, high-yielding, and possess desirable grain color and palatability, reflecting both production and consumption considerations. These preferences underscore the importance of demand-driven variety development tailored to the traits farmers value most, which can significantly enhance adoption and WTP for improved sorghum varieties. Empirical findings revealed significant heterogeneity in farmers’ WTP for improved sorghum varieties. Older farmers, those with larger landholdings, and those with access to non-farm income were generally more WTP compared to other farmer categories. The WTP curves were downward sloping and showed inelastic demand. This suggests that changes in price lead to proportionally smaller changes in the share of farmers’ WTP. Such inelasticity underscores the importance of non-price interventions, such as awareness, access, and tailored interventions targeting marginalized groups, such as youth and female farmers, to enhance the uptake of improved sorghum varieties.

We found gender disparity in WTP for improved sorghum varieties: only 40% of female-headed households were WTP the prevailing market price, compared to 60% of male-headed households. This gap highlights the importance of gender-responsive policy interventions to promote equitable access to improved agricultural technologies. To address these disparities, policymakers and development actors should consider implementing targeted subsidies or seed voucher schemes specifically for female-headed households. In addition, gender-inclusive extension services—including female-focused training and participatory demonstration plots—can help reduce sociocultural and institutional barriers that limit women’s adoption capacity. Making seeds and complementary inputs such as fertilizers available in smaller, more affordable package sizes (e.g., less than 5 kg bags) would also enhance access for resource-constrained female farmers, particularly those cultivating on small plots and having difficulty in transporting these inputs. By addressing both the affordability and access dimensions, such interventions can facilitate more uptake of improved sorghum varieties, contributing to inclusive agricultural transformation and gender equity.

Although these results are generated from seven districts from the two regional states of Ethiopia, we believe that having representative districts from the two major sorghum-producing regions supports the external validity of our findings to other moisture-stressed sorghum-producing areas that face similar social and environmental obstacles.

## Supporting information

S1 Data Data. (XLS)
